# Characterization of Parkinson's Disease Subtypes and Related Attributes

**DOI:** 10.3389/fneur.2022.810038

**Published:** 2022-05-23

**Authors:** Shamatree Shakya, Julia Prevett, Xiao Hu, Ran Xiao

**Affiliations:** ^1^School of Nursing, Duke University, Durham, NC, United States; ^2^School of Nursing, Emory University, Atlanta, GA, United States; ^3^Department of Biomedical Informatics, School of Medicine, Emory University, Atlanta, GA, United States; ^4^Department of Computer Science, College of Arts and Sciences, Emory University, Atlanta, GA, United States

**Keywords:** Parkinson's disease, symptoms, subtypes, cluster analysis, data driven approach

## Abstract

Parkinson's disease is a progressive neurodegenerative disease with complex, heterogeneous motor and non-motor symptoms. The current evidence shows that there is still a marked heterogeneity in the subtyping of Parkinson's disease using both clinical and data-driven approaches. Another challenge posed in PD subtyping is the reproducibility of previously identified PD subtypes. These issues require additional results to confirm previous findings and help reconcile discrepancies, as well as establish a standardized application of cluster analysis to facilitate comparison and reproducibility of identified PD subtypes. Our study aimed to address this gap by investigating subtypes of Parkinson's disease using comprehensive clinical (motor and non-motor features) data retrieved from 408 *de novo* Parkinson's disease patients with the complete clinical data in the Parkinson's Progressive Marker Initiative database. A standardized k-means cluster analysis approach was developed by taking into consideration of common practice and recommendations from previous studies. All data analysis codes were made available online to promote data comparison and validation of reproducibility across research groups. We identified two distinct PD subtypes, termed the severe motor-non-motor subtype (SMNS) and the mild motor- non-motor subtype (MMNS). SMNS experienced symptom onset at an older age and manifested more intense motor and non-motor symptoms than MMNS, who experienced symptom onset at a younger age and manifested milder forms of Parkinson's symptoms. The SPECT imaging makers supported clinical findings such that the severe motor-non-motor subtype showed lower binding values than the mild motor- non-motor subtype, indicating more significant neural damage at the nigral pathway. In addition, SMNS and MMNS show distinct motor (ANCOVA test: *F* = 47.35, *p*< *0.001*) and cognitive functioning (*F* = 33.93, *p*< *0.001*) progression trends. Such contrast between SMNS and MMNS in both motor and cognitive functioning can be consistently observed up to 3 years following the baseline visit, demonstrating the potential prognostic value of identified PD subtypes.

## Introduction

Parkinson's disease (PD) is a progressive neurodegenerative disease with a wide range of motor and non-motor manifestations (1). Motor features such as tremor, bradykinesia, rigidity, and postural instability are hallmarks for clinical assessment and diagnosis of PD (2). However, a growing body of evidence shows that PD is highly heterogeneous, including complex motor and non-motor features (3); and changes in brain imaging and cerebrospinal fluid (CSF) biomarkers (4–6). The detection, management, and assessment of PD can be incomplete and erroneous when non-motor features, imaging, and biospecimen changes are not considered. Both missed diagnosis and false diagnosis can severely affect the quality of life since misdiagnosis can result in undertreatment; thus, increasing the chance of rapid disease progression. Similarly, inaccurate diagnosis and treatment can expose individuals to serious side effects due to potent therapeutic medications (7). Given the heterogeneous nature of PD, it marks great clinical significance and benefits to identify underlying PD subtypes, which can be transformational in designing prevention and treatment strategies (8). Identifying subtypes of PD with shared characteristics can promote the discovery of PD etiology through the understanding of the underlying pathophysiological mechanism (1). In addition, the identification of subtypes opens the door for early detection and management of PD and the development of tailored management strategies, which help address the specific needs of an individual patient and prevent over/under treatment mishaps (8, 9).

Traditionally, motor symptoms are used for PD subtyping, with tremor-dominant (TD) and postural instability and gait difficulty (PIGD) as the most consistently identified motor subtypes (10, 11). These seminal studies provide crucial evidence on the existence of distinct subtypes of PD. However, non-motor features are grossly overlooked in the subtyping process. The fact that the choice of variables is driven by a priori hypothesis with the empirical section of cut-offs also introduces substantial ambiguity in the subtyping results (12). Since the first adoption of data-driven cluster analysis in PD subtyping (13), it has become a more popular approach in later studies in identifying PD subtypes. Cluster analysis can take a wide collection of variables into consideration for PD subtypes bypassing the a priori selection process, and determine the optimal subtypes based on statistical methods.

A body of literature exists utilizing cluster analysis with both motor and non-motor variables aiming to achieve an objective PD subtyping (13–18). Graham et al. identify three distinct PD subtypes, including “motor only,” “motor and cognition,” and “rapid progression” (13). Erro et al. also identify three PD subtypes but with different characteristics, with the first group bearing the lowest motor and non-motor burden, and the other two groups with a similar motor burden but different non-motor involvement (18). In another study, three distinct subtypes are defined as “mild motor-predominant,” “diffuse malignant,” and “intermediate” (17). Despite the positive findings, there are still wide inconsistencies in proposed subtypes of PD. One review paper compared subtyping results from two studies using the same database (17, 18) and found only 56% agreement on cluster memberships (4). A possible reason for these inconsistencies may be due to inherent limitations of data-driven subtyping of PD using hierarchical and non-hierarchical cluster analysis (6). The inconsistent subtypes can also stem from heterogeneity in data sources, study designs, patient populations (19), motor and non-motor data, and cluster analysis techniques used for identifying subtypes. The inclusion of PD patients on and off medications could be another source for inconsistencies in proposed PD subtypes (18); inclusion of PD patients on and off medications can also lead to erroneous PD subtyping. On top of inconsistent results, the poor reproducibility of reported PD subtypes poses another challenge in the field. Mestre et al. validated PD subtyping results from eight studies using the same database over a 10-year span by a panel of clinical experts following a modified Delphi consensus process (19). It was found that 7 out of 8 studies were “not very well” or “not at all” reproduced. Considering these issues in PD subtyping, multiple review papers outlined the need for standardized application of cluster analysis and additional results to reconcile the inconsistencies in PD subtyping (4, 12, 19).

This study aims to mitigate these issues by identifying PD subtypes in *de novo* PD patients using the comprehensive motor and non-motor data from the Parkinson's Progressive Markers Initiative (PPMI) (20). Following a common study pathway as summarized in (12), the present study starts off implementing data-driven k-means cluster analysis with a standardized approach using recommended practice in (6) to obtain objective PD subtyping. Next, the study conducts *post-hoc* analyses to describe motor, non-motor, CSF biomarkers and neuroimaging characteristics of PD subtypes. Lastly, the study evaluates the longitudinal progression of motor and global cognitive functioning of identified PD subtypes and the stability of these subtypes up to 3 years following the baseline visits. Our results are compared to previous studies to confirm past findings and to investigate discrepancies. Given that the data used in the study is publicly accessible, we also make our data analysis codes publicly available (https://github.com/ranxiao/PDSubtyping) to share the standardized cluster analysis approach, and maximally facilitate result comparison and reproducibility by other research groups.

## Materials and Methods

### Study Data and Population

Data used in this study were obtained from the PPMI database (20). PPMI is an ongoing longitudinal observational study involving participants from multiple clinical sites (21) situated in 11 countries. PMMI contains a wide range of information on demographics, motor, and non-motor features; Single-photon emission computed tomography (SPECT-DaT) brain imaging, cerebrospinal fluid (CSF) biomarker, and genetic biomarkers from three groups, namely (a) untreated *de novo* PD, (b) healthy control, and (c) SWEDD (scans without evidence of dopaminergic deficit) participants (20). We only included untreated *de novo* PD patients at the baseline to identify PD subtypes for this study. Clinical motor assessments were collected every 3 months during the first year and every 6-month interval after that. Assessments on cognitive and behavioral features were collected at every 12-month interval. Imaging data were collected from PD patients at visits in 12, 24, and 36 months (20). The details of the PPMI dataset and cohorts are available on the PPMI website www.ppmi-info.org. This study has received an exemption from the Institutional Review Board (IRB) of Duke University as it is using publicly available deidentified data from the PPMI.

The PPMI includes comprehensive information on motor and non-motor features from PD patients. Following data were used in our study:

1) Demographic characteristics: age of symptom onset and sex.
2) Motor features: International Parkinson's disease and Movement Disorder Society-Unified Parkinson's Disease Rating Scale (MDS-UPPRS) Part III total score and subscores: rigidity (item 3), tremor (sum of items 15–18), bradykinesia (sum of item 2, 4–9, and 14), and axial scores (sum of items 1 and 9–13) (22). Greater scores in motor features indicate greater severity of motor symptoms.
3) Non-motor features: Neuropsychiatric features—Montreal Cognitive Assessment (MoCA), psychological features from MDS-UPDRS Part I fatigue (item 13), pain (item 9), apathy (item 5), hallucinations (item 2), Geriatric Depression Scale (GDS), State of Anxiety Inventory (STAI) score, REM sleep behavior disorder (RBD); and autonomic features—olfactory dysfunction using the University of Pennsylvania Smell Identification Test (UPSIT) score, autonomic dysfunction using the Scales for Outcomes in Parkinson's disease Autonomic (SCOPA-AUT) total score and its sub item scores: orofacial, gastrointestinal, urinary, cardiovascular, thermoregulatory, pupilometer, and sexual scores. Greater scores in non-motor features indicate greater severity except MoCA and UPSIT scores.

We used the above-mentioned motor, non-motor data, and age of onset to identify Parkinson's disease subtypes and compared clinical attributes (motor, non-motor, brain imaging, CSF biomarkers, and symptom progressions) across the identified subtypes.

### Brain Imaging Markers

PPMI collected dopaminergic imaging—single-photon emission computed tomography (SPECT) from the PPMI participants. SPECT with CAT tracer I-ioflupane uses tracers that bind with dopamine cells, and SPECT imaging is a gold standard test to assess dopaminergic denervation in several neurodegenerative diseases, including Parkinson's disease (23, 24). A summary of imaging markers (right caudate, left caudate, right putamen, left putamen, right striatum, left striatum, mean caudate measure, mean putamen measure, mean striatum measure, lowest caudate measure, highest caudate measure, lowest putamen measure, highest putamen measure, lowest striatum measure, highest striatum measure, asymmetry index-caudate, putamen, striatum, left count density ratio, right count density ratio, contralateral striatum, contralateral caudate, contralateral putamen, contralateral count density ratio, ipsilateral striatum, ipsilateral caudate, ipsilateral putamen, ipsilateral count density ratio) was used to compare neurodegenerative imaging changes between subtypes at the baseline.

### CSF Biomarkers

PPMI includes comprehensive CSF biomarkers. Strong evidence underscores that CSF biomarkers can be sensitive indicators of cognitive decline (25), dopaminergic deficit (26), neurodegenerative changes (27), motor features (28), disease progression and neurofibrillary changes in PD (29). CSF biomarkers are found to be associated with motor progression, neuropsychiatric features, and cognitive decline in Parkinson's disease patients (30). We used a summary of CSF biomarkers (CSF Aβ1-42, α-synuclein, t tau, p-tau, t-tau/Aβ 1-42 ratio, p-tau/Aβ 1-42 ratio, p-tau/t-tau ratio, t-tau/α-synuclein ratio, p-tau/α-synuclein ratio) to compare CSF biomarkers across subtypes. Correlational analyses were also conducted to find the association of identified significant factors with motor and non-motor scores, respectively.

### Longitudinal Assessments

We compared the rate of motor and cognitive progression across the identified PD subtypes using Hoehn and Yahr's and MoCA scores. Patients included in the cluster analysis but without any of these symptom data in follow-up visits are identified and removed from the longitudinal assessments. Hoehn and Yahr's scale is demonstrated to have greater clinicometric properties and is highly correlated with the motor function of MDS-UPDRS (31). Hoehn and Yahr's scale has been used as a successful indicator of Parkinson's disease progression in clinical and clinical trial studies (32). The scores were assessed at the baseline (Y0), 12 months (Y1), 24 months (Y2), and 36 months (Y3) as described below in *post-hoc* analyses. A larger Hoehn and Yahr's score is an indication of severe motor dysfunction, whereas a lower MoCA score is reflective of greater cognitive impairment.

### K-Means Cluster Analysis

A total of 683 participants were in PPMI datasets, including Parkinson's disease, healthy control, and SWEDD (Scans without evidence of dopaminergic deficit). Among them, 423 untreated *de novo* participants with PD diagnosis at the baseline met the eligibility criteria for this study. After eliminating participants with missing values, 408 participants (males: 269, females: 139) with complete data were included for k-means cluster analysis. The average age of participants was 59.6 years (age range: 25–83 years). Twenty-two variables representing motor and non-motor features of PD were retrieved from PPMI datasets for cluster analysis. The corresponding clinical scores were standardized using min-max normalization. We conducted a k-means cluster analysis to identify the PD subtypes. A list of cluster numbers ranging from 2 to 10 was evaluated. The optimal number of subtypes was identified based on the Calinski-Harabasz (C-H) pseudo-F value. The cluster analysis was repeated 100 times with random initialization to avoid final cluster membership derived from a local optimum. The maximal number of iterations was set at 100 to ensure ample iterations to arrive at an optimal clustering solution within each cluster analysis. All analyses were carried out using Matlab (Mathworks Inc., Natick, MA).

### *Post-hoc* Analyses

The descriptive statistics (mean and standard deviation) were performed to summarize and compare the motor, non-motor features, SPECT-DaT brain imaging, and CSF biomarker attributes of two identified subtypes. Three *post-hoc* analyses were performed to (a) summarize motor and non-motor attributes, (b) SPECT-DaT brain imaging and CSF biomarkers, and (c) characterize motor and global cognitive progression rates, using Hoehn and Yahr and MoCA scores, respectively. The mean and standard deviation are calculated for each of the 22 baseline attributes and statistically compared between the two subtypes. The Hedges' g standardized mean difference (SMD) and its 95% confidence interval (*via* bootstrapping 3,000 times) are calculated to reveal the level of differences between subtypes. SMD is calculated by


(1) $$\begin{array}{r} SMD_{i}=\;\frac{m_{1i}-m_{2i}}{\delta_{i}},\;i=1,\ldots ,\;22 \end{array}$$

where *m*_1*i*_ and *m*_2*i*_ are the mean values of *i*^*th*^ baseline attribute in subtypes I and II, respectively. δ_*i*_ is the pooled standard deviation from both subtypes. The SMD is calculated using the measures of effect size (MES) toolbox ver.1.5 (33). The mean and standard deviation are calculated for each of 28 imaging measures and 10 CSF biomarkers, and statistically compared between the two subtypes. A non-parametric Wilcoxon rank-sum test was performed to examine if statistically significant differences existed between the identified subtypes in baseline clinical features (motor and non-motor features), imaging, and CSF biomarkers. The significant level α is set at 0.05, and multiple comparisons are adjusted by Bonferroni correction.

Visualizing cluster memberships is an important qualitative evaluation that can offer additional insights into derived PD subtypes. Due to the multivariate nature of input features, it's challenging to visualize the clusters in a two-dimensional format. While dimensional reduction techniques, such as principal component analysis (PCA), can be implemented to visualize the separation of different clusters (17), the subsequent interpretation is non-trivial contributed by the highly abstract nature of principal components. Like the global composite outcome proposed in (17), the present study establishes two new composite scores, the motor composite score, and the non-motor composite score. This is calculated by rescaling all motor and non-motor features (as described in the above section) into the range of (0, 1) through the min-max normalization and averaging the normalized scores of all components in each category, respectively. Since MoCA and UPSIT have revered severity scores compared to other non-motor features, they are adjusted by subtracting the normalized scores from one before the averaging operation for calculating the non-motor composite score. For both composite scores, the higher value indicates a severe condition. A scatter plot is generated to depict the distribution of cluster memberships of each sample in a two-dimensional plane representing the motor and non-motor symptom severity.

The progression is evaluated based on four time points, baseline visit (Y0), 12-month follow-up (Y1), 24-month follow-up (Y2), and 36-month follow-up (Y3). Three progression rates are derived to capture progression at various temporal resolutions, including early progression (change of scores between Y1 and Y0), secondary progression (change of scores between Y3 and Y1) and long-term progression (change of scores between Y3 and Y0). In addition, a time plot of motor and non-motor symptom progression is presented to qualitatively evaluate the stability of PD subtypes. An analysis of covariance (ANCOVA) test is performed to investigate the effect of PD subtypes and different visits on the symptom progression, which is adjusted with age at symptom onset as a covariate in the statistical analysis. The significant level α is set at 0.05.

## Results

### Baseline Description

A total of 408 *de novo* untreated PD patients comprising 269 (65.93%) males and 139 (34.96%) females with complete clustering data were included in the k-means cluster analysis.

### Statistical Clusters

**Figure 1:**
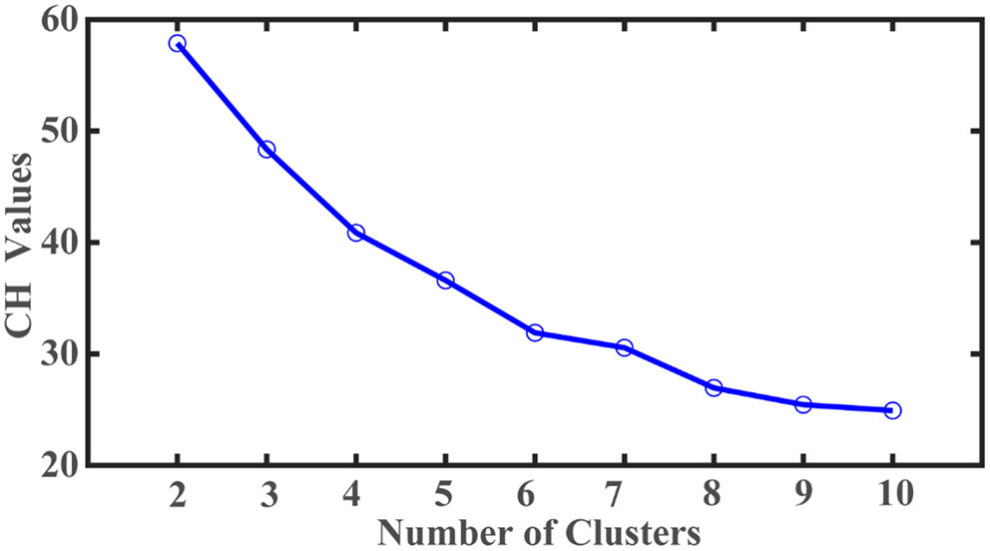
Selection of optimal cluster number based on Calanski-Harabasz (C-H) values.

There are twenty-two variables, including the age of symptom onset, motor, and non-motor features, used to identify PD subtypes based on the k-means cluster analysis. The clustering optimum was attained for the two-cluster solution. As shown in Figure 1, the largest C-H pseudo-F value obtained was 57 from the two-cluster solution. More samples are assigned to the PD subtype I (*N* = 270) than the second subtype (*N* = 138). PD subtype I includes participants who manifest symptoms at a younger age (young at symptoms onset) with an average age of 58.2 ± 10.2 (age range = 25.4–80.1 years) than PD subtype II participants (older at symptoms onset) with an average age of 62.4 ± 8.9 years (range = 35.6–83 years).

### Variation in Baseline Clustering Characteristics

**Figure 2:**
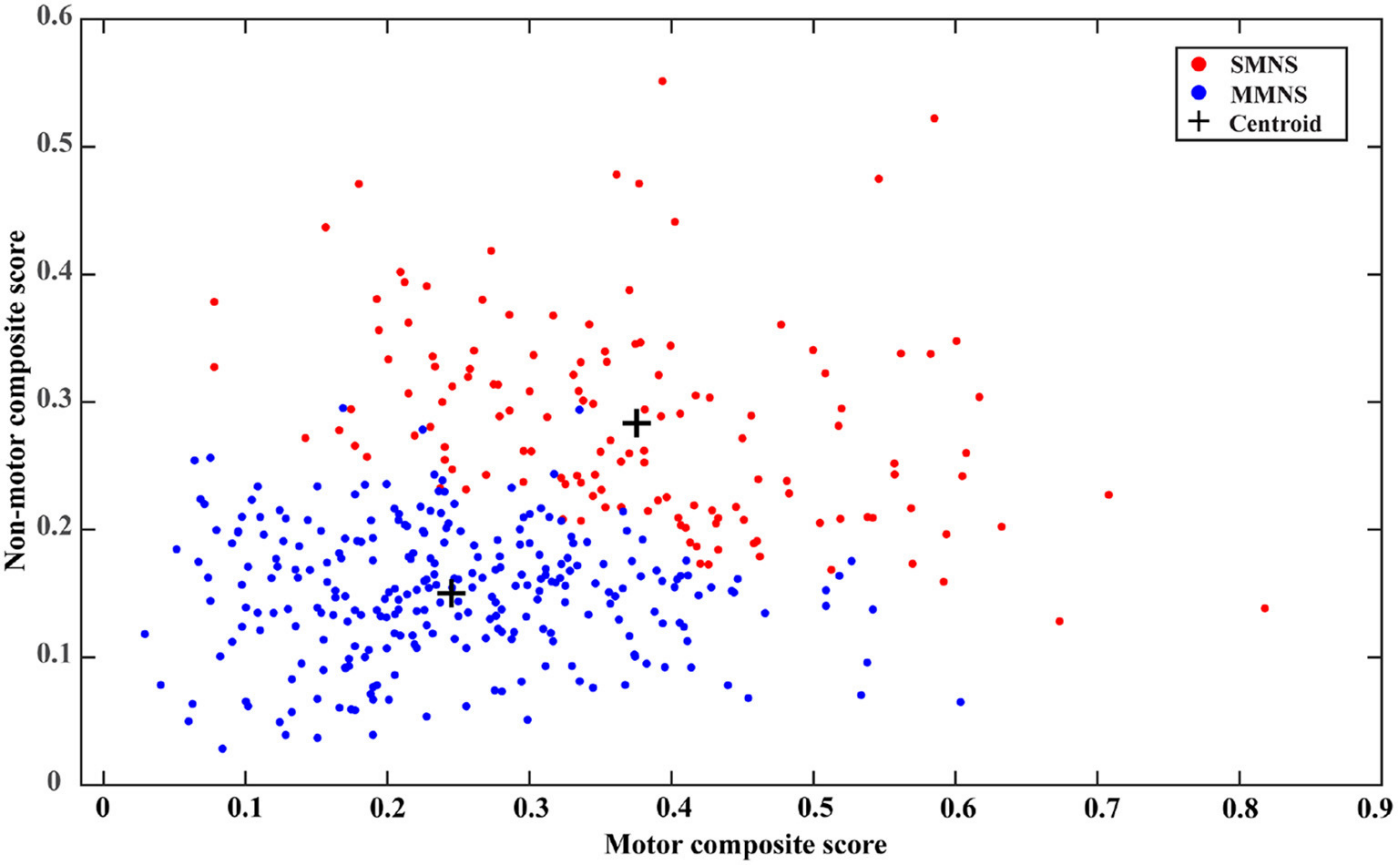
Distribution of cluster memberships in the motor-non-motor plane.

**Figure 3:**
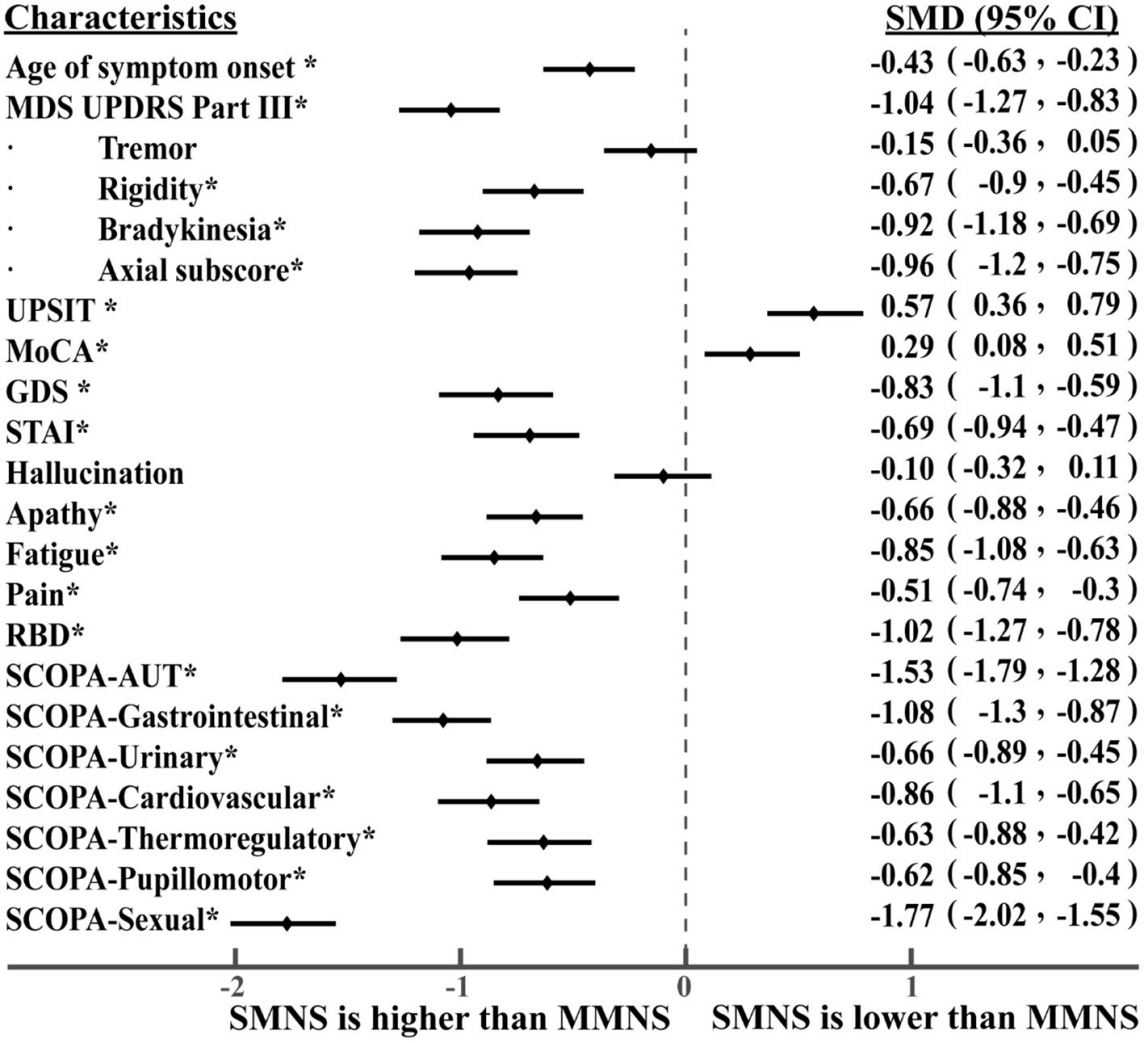
Level of differences in baseline characteristics between PD subtypes. The * symbol indicates statistically significant differences between the two subtypes.

As shown in Table 1, we observed statistically significant differences in a majority of motor and non-motor features. There were statistically significant differences in age of symptom onset (*p*< *0.001*), MDS UPDRS III score (*p*< *0.001*), rigidity (*p*< *0.001*), bradykinesia (*p*< *0.001*), axial subscore (*p*< *0.001*), MoCA (*p* = *0.0014*), GDS (*p*< *0.001*), fatigue (*p* < 0.001), pain (*p*< *0.001*), RBD (*p*< *0.001*), SCOPA-total (*p*< *0.001*), and SCOPA-subscores (*p*< *0.001*) between the two PD subtypes. No statistical significance was observed in their tremor (*p* = *0.1243*) and hallucination scores (*p* = *0.3408*). Overall, PD subtype II (older at symptom onset) experienced more severe Parkinson's symptoms (motor and non-motor symptoms) than the subtype I (younger at symptom onset). Subtype II experienced more severe cognitive impairment marked by a lower MoCA score than the subtype I. Based on the intensity of Parkinson's symptom burden, we termed the subtype I and the subtype II as “mild motor-non-motor subtype (MMNS)” and “severe motor-non-motor subtype (SMNS)”, respectively. Such observations are corroborated by the distribution of samples in the motor-non-motor plane, as presented in Figure 2. It shows SMNS samples (red dots) generally scatter on the top-right direction of MMNS samples (blue dots), with higher motor and non-motor composite scores. Figure 3 reveals the level of differences between the two subtypes with SMDs and corresponding 95% CIs. It shows that SMNS demonstrates more severe symptoms at baseline than the MMNS regardless of motor and non-motor attributes. The majority of attributes have a large effect size between the two subtypes, with most absolute SMD values close to one.

**Table 1:** Baseline clinical characteristics of PD subtypes.

**Characteristics**	**Mild motor and non-motor subtype** **(MMNS,** ***N* = 270)** **Mean (SD)**	**Severe motor and non-motor subtype** **(SMNS,** ***N* = 138)** **Mean (SD)**	***p*-value** **(α = 0.0022)**
Male sex (%)	177 (65.56%)	92 (66.67%)	-
**Age of symptom onset** ^*^	58.177 (±10.223)	62.363 (±8.899)	<0.001
MDS UPDRS Part III^*^	17.989 (± 7.278)	26.167 (±8.810)	<0.001
• Tremor	0.417 (±0.314)	0.465 (±0.314)	0.124
• Rigidity^*^	0.640 (±0.460)	0.981 (±0.583)	<0.001
• Bradykinesia^*^	0.708 (±0.372)	1.078 (±0.448)	<0.001
• Axial subscore^*^	0.248 (±0.283)	0.481 (±0.283)	<0.001
UPSIT^*^	23.830 (±7.842)	19.268 (±8.361)	<0.001
MoCA^*^	27.356 (±2.299)	26.696 (±2.316)	0.001
GDS^*^	1.682 (±1.997)	3.601 (±2.803)	<0.001
STAI^*^	61.226 (±15.289)	73.341 (±21.078)	<0.001
Hallucination	0.025 (±0.159)	0.043 (±0.204)	0.340
Apathy^*^	0.100 (±0.313)	0.413 (±0.681)	<0.001
Fatigue^*^	0.448 (±0.587)	1.073 (±0.965)	<0.001
Pain^*^	0.581 (±0.755)	1.007 (±0.956)	<0.001
RBD^*^	3.300 (±2.053)	5.739 (±2.960)	<0.001
SCOPA-AUT^*^	6.722 (±3.639)	15.145 (±6.387)	<0.001
SCOPA-gastrointestinal^*^	1.274 (±1.352)	3.840 (±2.169)	<0.001
SCOPA-urinary^*^	3.256 (±2.148)	6.167 (±3.536)	<0.001
SCOPA-cardiovascular^*^	0.300 (±0.554)	0.789 (±1.014)	<0.001
SCOPA-thermoregulatory^*^	0.800 (±1.072)	1.935 (±1.684)	<0.001
SCOPA-pupillomotor^*^	0.285 (±0.575)	0.681 (±0.715)	<0.001
SCOPA-sexual^*^	0.807 (±1.264)	1.732 (±1.878)	<0.001

* *Indicates statistical significance between the two subtypes (Wilcoxon rank-sum test)*.

### Variation in Imaging Markers

As shown in Table 2, SMNS and MMNS varied significantly in the brain imaging markers. Generally, the MMNS subtype demonstrated greater binding values in caudate, putamen, striatum regions than the SMNS subtype.

**Table 2:** Brain imaging of PD subtypes.

**Brain imaging**	**Mild motor and non-motor subtype** **(MMNS,** ***N* = 269)** **Mean (SD)**	**Severe motor and non-motor subtype** **(SMNS,** ***N* = 138)** **Mean (SD)**	***p*-value** **(α = 0.0017)**
Right caudate^*^	2.083 (±0.561)	1.841 (±0.601)	<0.001
Left caudate	2.060 (±0.563)	1.892 (±0.625)	0.001
Right putamen^*^	0.882 (± 0.350)	0.771 (±0.364)	<0.001
Left putamen	0.833 (±0.345)	0.776 (±0.375)	0.033
Right striatum^*^	2.966 (±0.844)	2.612 (±0.897)	<0.001
Left striatum	2.894 (±0.841)	2.668 (±0.954)	0.004
Mean caudate measure^*^	2.072 (±0.514)	1.866 (±0.574)	<0.001
Mean putamen measure^*^	0.858 (±0.279)	0.773 (±0.324)	<0.001
Mean striatum measure^*^	1.465 (±0.369)	1.319 (±0.425)	<0.001
Lowest caudate measure^*^	1.883 (±0.517)	1.692 (±0.539)	<0.001
Highest caudate measure^*^	2.261 (±0.542)	2.040 (±0.634)	<0.001
Lowest putamen measure^*^	0.691 (±0.247)	0.638 (±0.293)	<0.001
Highest putamen measure^*^	1.025 (±0.355)	0.909 (±0.387)	<0.001
Lowest striatum measure^*^	2.588 (±0.699)	2.341 (±0.783)	<0.001
Highest striatum measure^*^	3.271 (±0.836)	2.938 (±0.960)	<0.001
Asymmetry index (caudate)	19.047 (±13.143)	18.822 (±13.000)	0.854
Asymmetry index (putamen)	38.674 (±25.199)	34.826 (±24.438)	0.146
Asymmetry index striatum	23.549 (±14.47)	22.644 (±14.256)	0.529
Left count density ratio	2.707 (±1.027)	2.645 (±0.855)	0.854
Right count density ratio	2.547 (± 0.770)	2.619 (±0.890)	0.638
Contralateral striatum^*^	2.622 (± 0.717)	2.376 (±0.813)	<0.001
Contralateral caudate^*^	1.912 (±0.530)	1.710 (±0.561)	<0.001
Contralateral putamen	0.711 (±0.254)	0.665 (±0.300)	0.002
Contralateral count density ration	2.885 (±1.082)	2.758 (±0.787)	0.348
Ipsilateral striatum^*^	3.237 (±0.846)	2.904 (±0.956)	<0.001
Ipsilateral caudate^*^	2.232 (±0.547)	2.022 (±0.624)	<0.001
Ipsilateral putamen^*^	1.005 (±0.367)	0.882 (±0.397)	<0.001
Ipsilateral count density ratio	2.367 (±0.595)	2.507 (±0.805)	0.285

* *Indicates statistical significance between the two subtypes (Wilcoxon rank-sum test)*.

### Variation in CSF Biomarkers

The SMNS and MMNS subtypes did not vary significantly in terms of CSF biomarkers except the p-tau/α-synuclein ratio (*p* = 0.002), as shown in Table 3.

**Table 3:** CSF biomarkers of PD subtypes.

**CSF variables**	**Mild motor and non-motor subtype (MMNS,** ***N* = 246–266)** **Mean (SD)**	**Severe motor and non-motor subtype (SMNS,** ***N* = 115–133)** **Mean (SD)**	***p*-value** **(α = 0.005)**
Aβ1-42	920.070 (±384.060)	908.880 (±465.370)	0.529
α-synuclein	1,507.400 (±652.890)	1,519.400 (±713.130)	0.775
T-tau	166.100 (±50.812)	177.710 (±67.664)	0.260
P-tau	14.414 (±4.649)	16.006 (±6.296)	0.043
T-tau/Aβ1-42 ratio	0.1941 (±0.0822)	0.219 (±0.120)	0.080
P-tau/Aβ1-42 ratio	0.017 (±0.008)	0.019 (±0.012)	0.185
P-tau/T-tau ratio	0.085 (±0.006)	0.085 (±0.008)	0.624
Aβ 1-42/α-synuclein ratio	0.645 (±0.214)	0.645 (±0.307)	0.356
T-tau/α-synuclein ratio	0.114 (±0.026)	0.123 (±0.038)	0.058
P-tau/α-synuclein ratio^*^	0.009 (±0.003)	0.010 (±0.022)	0.002

* *Indicates statistically significant difference between two subtypes (Wilcoxon rank-sum test)*.

### Longitudinal Progression

**Figure 4:**
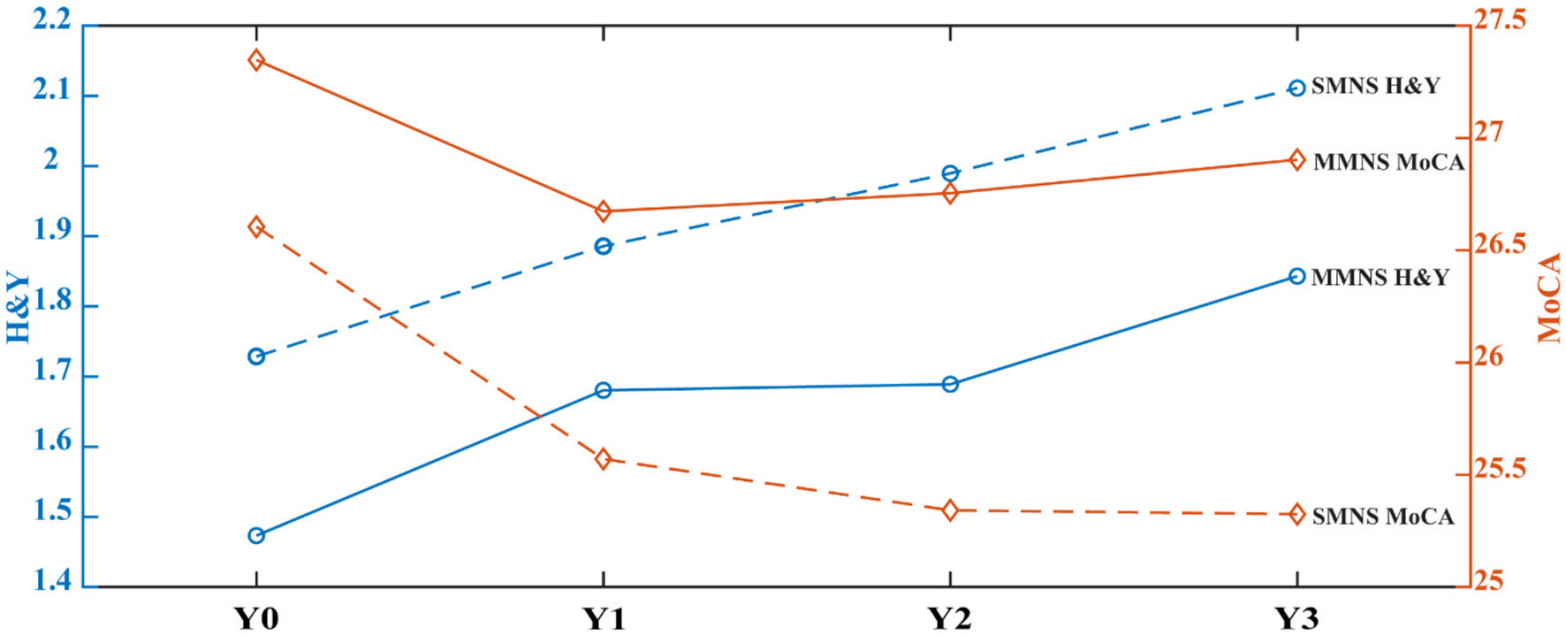
Time plot of motor (HandY) and non-motor (MoCA) progressions in PD subtypes.

There are 17 PD patients with baseline data but without symptom data in follow-up visits. This results in 391 patient data entering the longitudinal analysis with 262 in the MMNS group and 129 in the SMNS group, which maintain a similar patient ratio between two subtypes compared to those used in the cluster analysis. Figure 4 depicts the time plot of H&Y (blue lines) and MoCA (red lines) scores from baseline up to 36-month follow-up. The MMNS subtype is presented in solid lines and the SMNS subtype in dash lines. For the progression of the motor symptom as captured in H&Y (blue lines), SMNS presents a more severe pattern than MMNS consistently across all four time points. SMNS also presents fewer variations in the changing rate compared to MMNS. As for non-motor symptoms (red lines), SMNS presents worse cognitive functions than MMNS consistently across all four time points. It also shows an increasing difference in cognitive functions along time between the two subtypes. The ANCOVA test shows there is a significant difference between the two subtypes in both motor (*F* = 47.35, *p*< *0.001*) and non-motor (*F* = 27.67, *p*< *0.001*) progressions after adjusting age at symptom onset as a confounding factor. It also reveals significant difference among four timepoints in both motor (*F* = 33.93, *p*< *0.001*) and non-motor (*F* = 8.35, *p*< *0.001*) progressions.

Table 4 shows the comparison of the progression rates in three different time intervals between two subtypes. In terms of motor functioning, MMNS demonstrated more rapid early and long-term progression than the SMNS subtype. In terms of global cognitive function, measured using MoCA scores, patients in SMNS demonstrated rapid deterioration in early, secondary, and long-term progression than MMNS patients.

**Table 4:** Parkinson's disease symptom progression rate of PD subtypes.

**Characteristics**	**Mild motor and non-motor subtype (MMNS,** ***N* = 262)** **Mean (SD)**	**Severe motor and non-motor subtype** **(SMNS, *N* = 129)** **Mean (SD)**
**Hoehn and Yahr score**
- Early progression (Y1-Y0)	0.228 (±0.535)	0.171 (±0.528)
- Secondary progression (Y3-Y1)	0.169 (±0.587)	0.178 (±0.509)
- Long-term progression (Y3-Y0)	0.418 (±0.675)	0.383 (±0.699)
**MoCA score**
- Early progression (Y1-Y0)	−0.693 (±2.573)	−1.031 (±2.783)
- Secondary progression (Y3-Y1)	0.254 (±2.104)	−0.274 (±2.571)
- Long-term progression (Y3-Y0)	−0.579 (±2.798)	−1.210 (±2.882)

## Discussion

In the present study, we aimed to validate the Parkinson's disease subtypes using a standardized data-driven approach. We used k-means cluster analysis to unravel two distinct subtypes of Parkinson's disease patients using extensive motor and non-motor data from the Parkinson's Progression Markers Initiative (PPMI) database, which includes patients between 25 and 83 years, representing patients of broad age range and varying disease severity. We adopted a rigorous and standardized process to minimize biases in the cluster analysis, including data normalization, identifying optimal cluster numbers through an iterative process, and random initializations to avoid local optima. The process is fully data-driven without the need for manual parameter tuning such that objective and reproducible clustering results can be achieved. Based on the severity of motor and non-motor symptom burden, two distinct PD subtypes: severe motor-non-motor subtype (SMNS) and mild motor-non-motor subtype (MMNS) were identified. Novel motor and non-motor composite scores were developed to provide a direct and qualitative assessment of the distribution of cluster memberships. In addition, we provided a comprehensive evaluation of their characteristics, including brain imaging, CSF biomarkers, longitudinal motor and cognitive progression.

### Baseline Characteristics

Our *post-hoc* comparisons demonstrated that two subtypes varied significantly in terms of the age of symptoms onset, motor (MDS UPDRS III total score, rigidity, bradykinesia, and axial subscores) and non-motor (MoCA, SCOPA, RBD, GDS, pain, fatigue, and apathy) features. Although there is not much difference in the mean age of the two subtypes, the age range reflects the younger at symptom onset include patients of younger age (age range: 25.4–80.1 years) than older at symptom onset PD participants (age range: 35.6–83 years. These age ranges reflect the diversity in the age of *de novo* PD patients. The subtype with the older onset of symptoms experienced a greater Parkinson's symptoms burden than the subtype with the younger onset of symptoms. The SMNS subtype experienced more intense motor and non-motor symptoms than the MMNS subtype.

The severity of Parkinson's symptom in both groups were complemented by their respective SPECT imaging findings, which showed a marked reduction in binding values at caudate, putamen, and striatum regions in the SMNS relative to the MMNS subtype. These findings are in line with the earlier study findings, which demonstrated that reduced bindings values were indicative of Parkinson's disease (34). The intense Parkinson's symptom burden along with the shrinkage of caudate, putamen, striatum binding values in SMNS are suggestive of a pronounced dopaminergic neuronal loss in striatum nigral regions than MMNS groups (35). Earlier studies have indicated that greater loss of binding sites at caudate, putamen, and striatum regions were substantially correlated to impaired motor function, cognitive decline, and depression (21, 36, 37), which align with the current study findings.

Our findings from the CSF biomarkers comparison demonstrated that the two subtypes differ significantly regarding the p-tau/α-synuclein ratio. Our study is first to examine the correlations between the p-tau/α-synuclein ratio with MoCA and UPDRS-III scores. The analyses showed that p-tau/α-synuclein ratio was not significantly correlated with MoCA scores (Pearson correlation coefficient: ρ = 0.04, *p* = *0.50*) or UPDRS-III (Pearson correlation coefficient: ρ = 0.06, *p* = *0.23*). Despite insignificant findings, these findings suggest a critical need to examine the relationships between p-tau/α-synuclein ratio with MoCA and UPDRS-III scores in the long run, as the progression of PD patients might illustrate some meaningful relationships. Prior studies have shown that a lower p-tau/α-synuclein ratio is an important biomarker of PD and this ratio helps in differentiating PD from other neuropathies (30). However, the exact cut-off points of p-tau/α-synuclein ratios for differentiating PD patients are unclear because of conflicting findings. Even though our findings are not directly comparable with previous findings, prior studies comparing p-tau/α-synuclein ratios between PD patients and healthy cohorts show inconsistent findings. A higher p-tau/α-synuclein ratios were observed in PD patients relative to healthy controls (38), whereas another study showed that p-tau/α-synuclein ratios were not different between PD patients and healthy controls (39). It is important to note that the comparison groups of these studies vary from our PD subtypes, indicating the need to investigate the variation in p-tau/α-synuclein ratios across PD subtypes. A study using PPMI data indicated that the p-tau/α-synuclein ratios were not different across three identified subtypes of PD patients (17). Due to these discrepant findings, more studies are needed to investigate the role of the p-tau/α-synuclein ratio and variation of the p-tau/α-synuclein ratios across PD subtypes.

### Rate of Progression

Our findings on the rate of progression show that MMNS showed more rapid early and long-term deterioration of motor decline than SMNS as measured by Hoehn and Yahr score. In terms of global cognitive functioning, the SMNS group deteriorated more rapidly than MMNS patients did. Our findings confirmed the degenerative, progressive nature of Parkinson's disease in terms of both motor and cognitive domains. As depicted in Figure 4, clear contrast can be drawn in the progression of motor and non-motor symptoms between two subtypes. These findings shed light on unique trajectories of motor and cognitive dysfunctions in these two subtypes. Moreover, the ranking of severity in identified subtypes presents a stable pattern for both motor- and non-motor symptoms up to 3-year following the initial baseline visit, indicating the temporally consistent property of these subtypes, and demonstrating their potential prognostic value.

### Comparison With Previous Studies

Our findings are consistent with a study by Pagano et al. (40) using PPMI data with a priori age-based clusters. This study demonstrated that Parkinson's disease patients (older at symptom onset) manifested severe motor symptoms (tremor, MDS-UPDRS III total score, and bradykinesia), non-motor symptoms (SCOPA total, UPSIT, and MoCA), and marked deterioration in imaging makers than the younger at symptom onset (40). Our findings demonstrating the relationship between age of symptom onset and disease severity also agrees with previous studies by Fereshtehnejad et al. (17) and Erro et al. (18) based on data-driven analyses—without a priori hypotheses, using PPMI data. Especially, the SMNS subtype—that experienced more intense Parkinson's symptoms—bears some resemblance with their diffuse malignant subtype in respect of motor (MDS-UPDRS Part III score), non-motor (SCOPA-total, MoCA), and neuropsychiatric features (apathy, fatigue, pain, STAI). Our MMNS subtype with milder symptoms shares similarities with benign groups in terms of motor (MDS-UPDRS Part III score, tremor), non-motor (SCOPA-total, MoCA), and neuropsychiatric features (STAI, hallucinations, apathy, pain). Although the above-mentioned studies obtained their data from the common source, PPMI, findings reveal some discrepancies with respect to the motor and non-motor symptoms that might be due to the use of different clustering analysis techniques.

The past studies have reported that tremor is one of the key distinguishing features across various subtypes, whereas the current study showed that two subtypes did not vary in terms of tremor severity. Thus, our findings do not align with some past studies that identified unique subtypes with tremor and non-tremor dominant features (41, 42). There are several plausible reasons explaining differences in our findings with these studies. One reason could be that most of these studies utilized motor data alone, while our study considered both motor and non-motor symptoms data of the PD. The second plausible reason might be due to consideration of cross-sectional tremor data in the cluster analysis that might have prevented tracking of tremor progression and discrepancies in identified subtypes. Finally, it might be because of PPMI data as PPMI only includes *de novo* PD patients who are off the medications, which might have excluded the PD who were on medications with greater tremor severity. The disease progression rates in this current study were also inconsistent with prior studies, which report that the old-age onset subtype showed rapid motor decline than the young-onset subtype having slower progression of motor decline (41, 42). The likely reasons for these inconsistencies can be due to differences in variables included in cluster analyses, data pre-processing, data standardization, and clustering techniques applied for identifying clusters.

### Strengths and Limitations

The present study developed a standardized cluster analysis procedure that builds upon recommendations and common practice from previous studies (4, 6, 12, 18), and it is published online to foster data comparison and reproducibility of results. We undertook measures to mitigate the inherent limitations associated with data-driven approach analysis: (a) inclusion of comprehensive motor and non-motor features from the publicly available PPMI database, (b) data standardization using min-max normalization, (c) identification and selection of optimum clusters using C-H pseudo-F value, (d) identification of global minima and avoidance of local minima by setting iterations and repetitions to 100 levels. These measures aid in minimizing any subjective biases that might have stemmed from a priori hypothesis-driven analysis, clinical subtyping, and the heuristic selection of variables and clusters. Our study explored and compared attributes of two clusters using motor and non-motor features, brain imaging, CSF-biomarkers, trajectories of motor, and global cognitive functioning. The novel composite scores combined with visualization of cluster memberships in the two-dimensional motor-non-motor plane provide a more intuitive and clinically meaningful understanding of identified PD subtypes. In addition, few studies have investigated the longitudinal stability of subtype characteristics. Our results show a clear and stable contrast between the two subtypes in both motor and cognitive progressions. Such stability provides not only additional validation but also offers great clinical implications in terms of the prognostic value of the identified PD subtypes.

The last strength of the study inherits from the advantage of the data-driven cluster analysis in that once clusters have been identified, a rule for assigning cluster memberships (i.e., PD subtypes in this study) is readily constructed and can be used to classify patient data into one of these two PD subtypes. Our K-means cluster analysis approach derives rescaling parameters for normalizing patient data and centroid information for the two PD subtypes (see Supplementary Table S1). These parameters collectively form the cluster model, which can classify patients into one of two PD subtypes based on the following steps, (a) normalize patient data using rescaling parameters; (b) calculate squared Euclidean distances between patient data and each cluster centroid; (c) classify the patient into the PD subtype with a shorter distance. The simple implementation and explainable model parameters facilitate the downstream clinical applications of identified PD subtypes. Compared to traditional clinical subtyping that is usually based on a priori hypothesis and relies on motor symptoms and/or the age of onset (10, 11, 43–46), data-driven PD subtyping addresses some of the limitations. This includes incorporating important non-motor features in the subtyping process and bypassing the ambiguity in selecting the ratio of motor sub-scores for defining the clinical subtypes (12, 47). However, several challenges still exist, staggering the broad adoption of data-driven subtypes. First, data-driven PD subtyping often requires more data elements to be collected, which imposes an additional burden in clinical practice. Second, there is still a substantial variation between subtypes identified from different data-driven PD subtyping studies. Our study answers the call for a standardized cluster analysis approach (12) and facilitates result sharing and comparison. More efforts need to be directed in this direction to identify the most relevant variables and consolidate discrepant findings for data-driven PD subtyping to push their clinical use.

Despite the strengths in the study, our findings should be interpreted with some degree of caution partially due to limitations associated with PPMI datasets. PPMI involves untreated PD patients at the early PD stage without extreme motor or cognitive complications. This might underrepresent patients with greater symptoms severity who are on medications. PPMI has a shorter follow-up duration (<4 years); thus, it was not possible to examine the long trajectories of disease severity across subtypes. Furthermore, confounding factors in longitudinal analyses in observational studies often exist. Although the issue is mitigated by including the age at symptom onset as a covariate in the ANCOVA test, other potential confounding factors have yet to be explored due to the limitations of existing datasets. For example, procedures such as deep brain stimulation (DBS) are likely to alter the symptom progression trajectories. However, among 391 patient data entered the longitudinal analysis, only 48 of them have information on whether and when DBS procedures are administrated. Therefore, future study is warranted to investigate the confounding effects further when more complete datasets become available.

Although PPMI includes patients of a wider age range and disease severity, we chose to exclude the participants with any missing values in variables used for cluster analysis to prevent potential biases that might be introduced due to the replacing of missing values. Due to greater missing values in imaging and CSF biomarkers, several participants were excluded from analyses that might have compromised the generalizability of findings. This exclusion of participants with missing values might have restricted the representativeness of the PPMI dataset. The inclusion of participants with complete data for cluster analysis might have reduced the strength of the data-driven analysis due to the truncation of the analytic sample size. In addition, we only summarize clinical, brain imaging, and CSF biomarker characteristics of two subtypes using descriptive measures; thus, this might not explain the correlation between clinical, imaging, and biomarker changes.

As the PPMI dataset is still evolving with the inclusion of motor and non-motor features and longer follow-up duration, further confirmatory studies are required to validate PD subtypes. Identification of subtypes of PD patients using the latest, complete PPMI data will help in the precise identification of PD subtypes representing PD patients. Furthermore, subtypes can be identified using brain imaging, biomarkers, and genetics data. Moreover, future studies need to examine the correlation between clinical features, CSF, and imaging markers to elucidate the complex heterogeneous nature of Parkinson's disease. Other clustering approaches, such as network analysis, can be used to validate PD subtypes.

## Conclusion

In summary, we identified two distinct subtypes of Parkinson's disease: (a) mild motor-non-motor subtype (MMNS) with milder motor, non-motor, and neuropsychiatric features and (b) severe motor-non-motor subtype (SMNS) with intense motor and non-motor features by including motor and non-motor features in cluster analysis. The SPECT imaging supports the clinical symptom burden—lower binding values in striatal pathways were observed in SMNS than MMNS, reflecting greater deterioration in nigral pathways in the SMNS subtype. Both subtypes showed peculiar progression patterns, with SMNS showing a more rapid decline in global cognitive functioning and MMNS showing a more rapid decline in motor functioning. The clear contrast between two subtypes in both motor and cognitive functioning progressions demonstrates the longitudinal stability of subtype memberships up to 3 years following the baseline visit. Such stability provides great clinical implications on the prognostic value of the identified subtypes at baseline. More studies are needed to confirm our findings with a larger sample of longitudinal data for subtyping analysis. Such findings provide additional evidence to devise tailored treatment planning for identified PD subtypes to maximize patient outcomes.

## Data Availability Statement

The data analyzed in this study was obtained from the Parkinson's Progressive Marker Initiative (PPMI; https://www.ppmi-info.org/), the following licenses/restrictions apply: Investigators seeking access to PPMI data must submit an Online Application, sign the Data Use Agreement and comply with the study Publications Policy. Requests to access these datasets should be directed to the PPMI Data and Publications Committee (DPC), https://www.ppmi-info.org/access-data-specimens/download-data.

## Author Contributions

RX and XH contributed to the conception, design of the work, revised the work, and have approved the submitted version. SS, JP, and RX contributed to the analysis and interpretation of the data. SS drafted the work. All authors contributed to the article and approved the submitted version.

## Conflict of Interest

The authors declare that the research was conducted in the absence of any commercial or financial relationships that could be construed as a potential conflict of interest.

## Publisher's Note

All claims expressed in this article are solely those of the authors and do not necessarily represent those of their affiliated organizations, or those of the publisher, the editors and the reviewers. Any product that may be evaluated in this article, or claim that may be made by its manufacturer, is not guaranteed or endorsed by the publisher.
