# Supplementary material for: Characterization of Parkinson's Disease Subtypes and Related Attributes
Source: Front Neurol. 2022 May 23;13:810038. doi: 10.3389/fneur.2022.810038 (PMC9167933; doi:10.3389/fneur.2022.810038)
Supplement: Supplementary file 1 [file Table_1.DOCX]

**Table S1.** Parameters for cluster membership assignment

| **Clinical Variables** | **Rescaling Parameters** | | **Cluster Centroids** | |
| --- | --- | --- | --- | --- |
|  | **Center factor** | **Scaling factor** | **MMNS** | **SMNS** |
| **Age of symptom onset** | 25.37 | 57.64 | 0.57 | 0.64 |
| **MDS UPDRS Part III** | 4 | 47 | 0.30 | 0.47 |
| - **Tremor** | 0 | 1.8 | 0.23 | 0.26 |
| - **Rigidity** | 0 | 2.6 | 0.25 | 0.38 |
| - **Bradykinesia** | 0.08 | 2.23 | 0.28 | 0.45 |
| - **Axial** | 0 | 1.5 | 0.17 | 0.32 |
| **UPSIT** | 1 | 39 | 0.59 | 0.47 |
| **MoCA** | 17 | 13 | 0.80 | 0.75 |
| **GDS** | 0 | 14 | 0.12 | 0.26 |
| **STAI** | 40 | 97 | 0.22 | 0.34 |
| **Hallucination** | 0 | 1 | 0.03 | 0.04 |
| **Apathy** | 0 | 4 | 0.03 | 0.10 |
| **Fatigue** | 0 | 4 | 0.11 | 0.27 |
| **Pain** | 0 | 4 | 0.15 | 0.25 |
| **RBD** | 0 | 12 | 0.28 | 0.48 |
| **SCOPA-AUT** | 0 | 39 | 0.17 | 0.39 |
| **SCOPA-Gastrointestinal** | 0 | 11 | 0.12 | 0.35 |
| **SCOPA-Urinary** | 0 | 18 | 0.18 | 0.34 |
| **SCOPA-Cardiovascular** | 0 | 6 | 0.05 | 0.13 |
| **SCOPA-Thermoregulatory** | 0 | 7 | 0.11 | 0.28 |
| **SCOPA-Pupillomotor** | 0 | 3 | 0.10 | 0.23 |
| **SCOPA-Sexual** | 0 | 6 | 0.13 | 0.29 |
